# Reproducibility of the external surface position in left‐breast DIBH radiotherapy with spirometer‐based monitoring: methodological mistake

**DOI:** 10.1120/jacmp.v15i4.4909

**Published:** 2014-07-08

**Authors:** Siamak Sabour

**Affiliations:** ^1^ Safety Promotion and Injury Prevention Research Center, and Department of Clinical Epidemiology School of Health Shahid Beheshti University of Medical Sciences Tehran Iran

Dear Editor,

I was interested to read the papers by Fassi and colleagues, published in the January 2014 issue of *Journal of Applied Clinical Medical Physics* (JACMP).^(1)^ The authors aimed to evaluated the reproducibility of deep inspiration breath hold (DIBHs) controlled by a spirometric device, by assessing the variability of the external surface position within a single DIBH (intra‐DIBH) and between DIBHs performed in the same treatment session (intrafraction) or in different sessions (interfraction).^(1)^

As the authors pointed out, displacements of the external surface between different sessions were up to 6.3 mm along a single direction, even at constant inspired volumes. The median value of the interfraction variability in the position of breast passive markers was 2.9 mm (range 1.9–4.8 mm) in the latero–lateral direction, 3.6 mm (range 2.2–4.6 mm) in the antero–posterior direction, and 4.3 mm (range 2.8–6.2 mm) in the cranio–caudal direction.^(1)^ Such descriptive results has nothing to do with reliability analysis.^2^, ^3^, ^4^, ^5^, ^6^, ^7^, ^8^ Why did the authors not used well‐known tests for reliability, such as intraclass correlation coefficient (ICC) or weighted kappa?^2^, ^3^, ^4^, ^5^, ^6^, ^7^, ^8^

Regarding reliability or agreement, it is good to know that ICC should be used for quantitative variables and weighted kappa (not simple kappa, because kappa has its own limitations, too) for qualitative ones.^2^, ^3^, ^4^, ^5^, ^6^, ^7^, ^8^ Moreover, Fassi and colleagues reported no significant dose distribution variations in their study.^(1)^ It is crucial to know that statistically significant is completely different from clinically importance, and should not be confused with each other. Moreover, statistics cannot provide a simple substitute for clinical judgment.^2^, ^3^, ^4^, ^5^, ^6^, ^7^, ^8^

As the authors pointed out in their conclusion, spirometer‐based control does not guarantee a reproducible position of the external surface in left‐breast DIBH radiotherapy. Such a conclusion is simply a misinterpretation, due simply to inappropriate use of statistical test.

## Supporting information

Supplementary Material
