# Supplementary material for: Reproducibility of the external surface position in left‐breast DIBH radiotherapy with spirometer‐based monitoring: methodological mistake
Source: J Appl Clin Med Phys. 2014 Jul 8;15(4):400. doi: 10.1120/jacmp.v15i4.4909 (PMC5875527; doi:10.1120/jacmp.v15i4.4909)
Supplement: Supplementary file 1 — Supplementary Material [file ACM2-15-400-s001.doc]

[**Reproducibility of the external surface position in left-breast DIBH radiotherapy with spirometer-based monitoring;**](http://www.ncbi.nlm.nih.gov/pubmed/24423845) **Methodological Mistake**

Siamak Sabour, MD, MSc, DSc, PhD, Postdoc ^1,2^

^1^ Safety Promotion and Injury Prevention Research Center, Shahid Beheshti University of Medical Sciences, Tehran

I.R. Iran

^2^ Department of Clinical Epidemiology, School of Dentistry, Shahid Beheshti University of Medical Sciences, Tehran, I.R. Iran

**Corresponding contributor:** Siamak Sabour

Safety Promotion and Injury Prevention Research Center, Shahid Beheshti University of Medical Sciences, Tehran

I.R. Iran

E: [s.sabour@sbmu.ac.ir](mailto:s.sabour@sbmu.ac.ir)

T: +98-21- 22421814

Type of article: Letter to the editor

Total number of pages:1

Total number of photographs:0

Source(s) of support: None

Support: No

Conflicts of interest: No

Permissions: not relevant

**Advances in Knowledge:**

1. The reliability is being assessed by inappropriate tests.

**Implication for patient care:**

Misdiagnosis and mismanagement of the patients in routine clinical care cannot be avoided using inappropriate tests to assess reliability

[**Reproducibility of the external surface position in left-breast DIBH radiotherapy with spirometer-based monitoring;**](http://www.ncbi.nlm.nih.gov/pubmed/24423845) **Methodological Mistake**

I was interested to read the papers by Fassi A and colleagues published in Jan 2014 issue of [*J Appl Clin Med Phys.*](http://www.ncbi.nlm.nih.gov/pubmed)^1^ The authors aimed to evaluated the reproducibility of Deep inspiration breath hold (DIBHs) controlled by a spirometric device, by assessing the variability of the external surface position within a single DIBH (intra-DIBH) and between DIBHs performed in the same treatment session (intrafraction) or in different sessions (interfraction).^1^ As the authors pointed out displacements of the external surface between different sessions were up to 6.3mm along a single direction, even at constant inspired volumes. The median value of the interfraction variability in the position of breast passive markers was 2.9mm (range 1.9-4.8 mm) in the latero-lateral direction, 3.6 mm (range 2.2-4.6mm) in the antero-posterior direction, and 4.3mm (range 2.8-6.2 mm) in the cranio-caudal direction.^1^ Such descriptive results has nothing to do with reliability analysis. ^2-8^ Why did the authors not used well known tests for reliability such as Intra Class Correlation Coefficient (ICC) or weighted kappa? ^2-8^ Regarding reliability or agreement, it is good to know that ICC should be used for quantitative variables and weighted kappa (not simple kappa because kappa has its own limitations too) for qualitative ones. ^2-8^ Moreover, they reported no significant dose distribution variations in their study.^1^ It is crucial to know that statistically significant is completely different with clinically importance and should not be confused with each other. Moreover, statistics cannot provide a simple substitute for clinical judgment. ^2-8^ As the authors pointed out in their conclusion, spirometer-based control does not guarantee a reproducible position of the external surface in left-breast DIBH radiotherapy. Such a conclusion is simply a misinterpretation just due to inappropriate use of statistical test.

**References:**

1. [*Fassi A*](http://www.ncbi.nlm.nih.gov/pubmed?term=Fassi A%5BAuthor%5D&cauthor=true&cauthor_uid=24430704)*,* [*Ivaldi GB*](http://www.ncbi.nlm.nih.gov/pubmed?term=Ivaldi GB%5BAuthor%5D&cauthor=true&cauthor_uid=24430704)*,* [*Meaglia I*](http://www.ncbi.nlm.nih.gov/pubmed?term=Meaglia I%5BAuthor%5D&cauthor=true&cauthor_uid=24430704)*,* [*Porcu P*](http://www.ncbi.nlm.nih.gov/pubmed?term=Porcu P%5BAuthor%5D&cauthor=true&cauthor_uid=24430704)*,* [*Tabarelli de Fatis P*](http://www.ncbi.nlm.nih.gov/pubmed?term=Tabarelli de Fatis P%5BAuthor%5D&cauthor=true&cauthor_uid=24430704)*,* [*Liotta M*](http://www.ncbi.nlm.nih.gov/pubmed?term=Liotta M%5BAuthor%5D&cauthor=true&cauthor_uid=24430704)*,* [*Riboldi M*](http://www.ncbi.nlm.nih.gov/pubmed?term=Riboldi M%5BAuthor%5D&cauthor=true&cauthor_uid=24430704)*,* [*Baroni G*](http://www.ncbi.nlm.nih.gov/pubmed?term=Baroni G%5BAuthor%5D&cauthor=true&cauthor_uid=24430704)*.* [*Reproducibility of the external surface position in left-breast DIBH radiotherapy with spirometer-based monitoring,*](http://www.ncbi.nlm.nih.gov/pubmed/24423845) [*J Appl Clin Med Phys.*](http://www.ncbi.nlm.nih.gov/pubmed) *2014 Jan 4;15(1):4494. doi: 10.1120/jacmp.v15i1.4494.*
2. *Jeckel. J.F, Katz. D.L, Elmore, J.G, Wild, D.M.G, Epidemiology, Biostatistics and Preventive Medicine, 3rd edition. 2007, SAUNDERS, Elsevier, Philadelphia, PA, United State*
3. *Sabour S, Dastjerdi EV.* [*Reliability of four different computerized cephalometric analysis programs: a methodological error.*](http://www.ncbi.nlm.nih.gov/pubmed/24132404)*Eur J Orthod. 2013 Dec;35(6):848. doi: 10.1093/ejo/cjs074. Epub 2013 Oct 16.*
4. *Sabour S, Moezizadeh M, Dastjerdi EV.* [*Reliability of shade selection using an intraoral spectrophotometer: common mistakes in reliability analysis.*](http://www.ncbi.nlm.nih.gov/pubmed/23404557) *Clin Oral Investig. 2013 Apr;17(3):1025. doi: 10.1007/s00784-013-0930-6. Epub 2013 Feb 13.*
5. *Sabour S, Dastjerdi EV.* [*Reliability of assessment of nasal flow rate for nostril selection during nasotracheal intubation: common mistakes in reliability analysis.*](http://www.ncbi.nlm.nih.gov/pubmed/23333213) *J Clin Anesth. 2013 Mar;25(2):162. doi: 10.1016/j.jclinane.2012.10.006. Epub 2013 Jan 16.*
6. *Sabour S.* [*Reliability and repeatability of toe pressures measured with laser Doppler and portable and stationary photoplethysmography devices.*](http://www.ncbi.nlm.nih.gov/pubmed/23068429) *Ann Vasc Surg. 2012 Nov;26(8):1167. doi: 10.1016/j.avsg.2012.05.008.*
7. *Lawrence I, Kuei Lin, A Concordance Correlation Coefficient to Evaluate Reproducibility, BIOMETRICS, 1989, March, 45, 255-268*
8. *Sabour S, Ghassemi F.* [*The reproducibility of measurements of differential renal function in paediatric 99mTc-MAG3 renography: is this correct?*](http://www.ncbi.nlm.nih.gov/pubmed/22972373) *Nucl Med Commun. 2012 Dec;33(12):1311; author reply 1311-2. doi:10.1097/MNM.0b013e328359453a.*
